# Switching Drugs for Livestock May Help Save Critically Endangered Asian Vultures

**DOI:** 10.1371/journal.pbio.0040061

**Published:** 2006-01-31

**Authors:** Liza Gross

In just ten years, tens of millions of vultures have vanished from the Indian subcontinent. Since the early 1990s, white-backed *(Gyps bengalensis)*, long-billed *(G. indicus)*, and slender-billed *(G. tenuirostris)* vulture populations have dropped by over 95%. In Europe, it was clear that human persecution eradicated bearded and griffon vultures from some countries (reintroduction and protection efforts are now restoring populations). But in India, where it is illegal to kill wildlife and the bird is valued for its ecological role, their unprecedented decline was puzzling. Dead birds found in India, Pakistan, and Nepal had extensive visceral gout (a buildup of uric acid crystals in the internal organs associated with renal failure). The birds often appeared sick and lethargic, some showed prolonged severe neck drooping, before collapsing—sometimes from their perches.

Intensive testing failed to implicate infectious disease, pesticide poisoning, starvation, and other possible causes. Then, finally, in 2004 a team of scientists from the United States–based Peregrine Fund made a breakthrough. In the mid-1990s, livestock farmers in India began treating their cattle and water buffaloes with the nonsteroidal anti-inflammatory drug (NSAID) diclofenac—a known kidney toxin in mammals. Vultures, it turned out, were highly sensitive to the drug, which they ingested while feeding at carcass dumps, the traditional method of livestock disposal in South Asia. Diclofenac later came into widespread use in Pakistan and Nepal. A subsequent concentrated research effort demonstrated that diclofenac use was on a sufficient scale to fully account for the declines. As a consequence, the Indian government announced its intention to ban veterinary use of the drug in March, but progress has been frustrated in part by the lack of a safe yet effective alternative. In a new study, toxicologist Gerry Swan and a team of colleagues from South Africa, Namibia, India, and the United Kingdom show that they have found that alternative.

The consequences of the vulture collapse have already reverberated across the subcontinent. Americans and Europeans once persecuted vultures, thinking they transmitted disease, but vultures help control brucellosis, anthrax, and other livestock diseases by consuming infected carcasses. In their absence, feral dog populations have exploded, likely increasing the risk of human attacks and the spread of rabies. If rats follow suit, bubonic plague and other rodent-transmitted diseases may also increase. And because these scavengers can't match vultures' efficiency as flesh-eaters, many carcasses—including human—lay rotting. In sky burials, Zoroastrian Parsis (and Tibetans, in a slightly different ritual) leave their dead on platforms for vultures to devour, to avoid defiling earth, water, or fire with an unholy corpse. Where corpses once attracted 300 vultures—which could pick a body clean in half an hour—today so few remain that many Parsis must find new ways to send off their dead.

To find an anti-inflammatory that could treat livestock without killing vultures, Swan et al. collected records on NSAID use and effects on captive *Gyps* vultures from veterinarians at zoos and bird of prey collections around the world. They settled on meloxicam—the only NSAID that had been used extensively on vultures with no evidence of kidney damage—as a promising candidate. They first tested the drug's safety on a species that faces no risk of extinction, but suffers the same diclofenac toxicity as its endangered brethren: the African white-backed vulture *(G. africanus)*.

The six-phase safety trial was designed to minimize experimental birds' suffering and risk of death. In the first three phases, five vultures orally received meloxicam through a tube; three controls received water by the same method. After ensuring the health of all the birds by analyzing blood levels of uric acid and other markers, the authors increased the dose for the next phase. By the third phase, the dose just exceeded the estimated maximum likely exposure for wild vultures.

Though the difference in mortality risk between meloxicam and diclofenac was statistically significant—all vultures in Phases I–III survived meloxicam treatment while both *G. africanus* vultures treated with diclofenac in a previous study died—the small sample size can't preclude all risk. But strong corroborating evidence of safety comes from comparing blood samples of treated and control birds from both studies: diclofenac-treated vultures had a marked and dose-dependent elevation of uric acid levels compared to controls; meloxicam-treated vultures showed no such differences.

The authors next expanded the number of birds receiving the highest dose of meloxicam, treating 11 captive and 21 wild *G. africanus* vultures (plus captive and wild birds as controls). The wild birds were captured on a special expedition to Namibia, held in temporary facilities, and released after the experiment. All the vultures survived and showed no changes in blood uric acid levels. In the fifth phase, captive *G. africanus* vultures ate liver and muscle tissue from cattle treated with above-standard doses of meloxicam, to mimic the natural route of exposure and account for the possibility that treated cattle might produce toxic metabolites. Again, all survived without elevated uric acid levels or ill effects. As a final test, the authors treated ten endangered Asian vultures of two species with meloxicam; five received the maximum likely exposure. All ten were alive and healthy four months after the treatment.

These results make a strong case that the recovery of the Asian vulture depends on immediate action to replace diclofenac with meloxicam. The authors hope other researchers use this approach to evaluate the safety of veterinary drugs on vultures and other scavengers—preferably before the drugs reach the market. For more information, go to Darwin Initiative, http://www.darwin.gov.uk/projects/details/10013.html; Vulture Rescue, http://www.vulturedeclines.org; and BirdLife International, http://www.birdlife.org/action/science/species/asia_vulture_crisis/index.html.

## 

**Figure:**
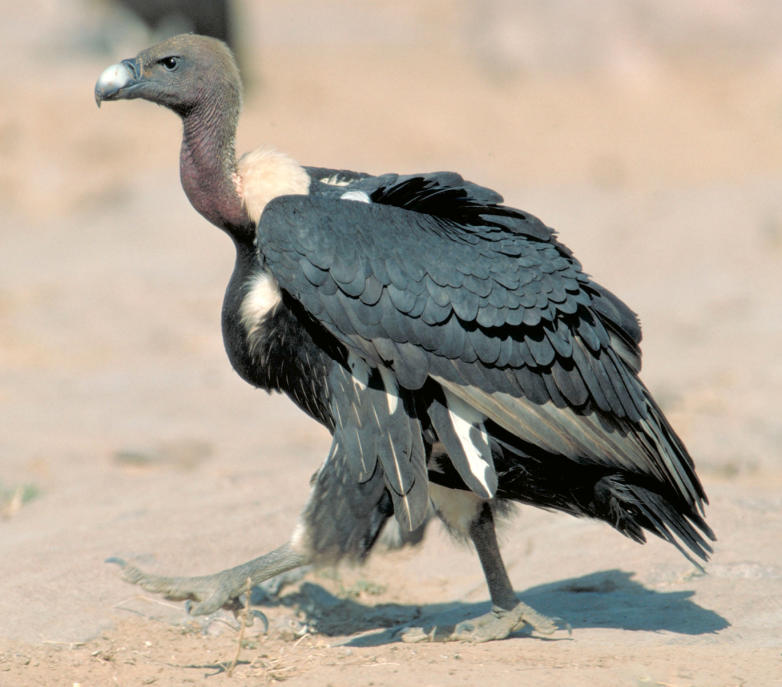
Tens of millions of Asian vultures–including the oriental whitebacked vulture (Gyps bengalensis) , above–have vanished from the Indian subcontinent since the early 1990s. (Image: Goran Ekstrom)

